# Analysis of homeobox gene action may reveal novel angiogenic pathways in normal placental vasculature and in clinical pregnancy disorders associated with abnormal placental angiogenesis.

**DOI:** 10.3389/fphar.2014.00133

**Published:** 2014-06-04

**Authors:** Padma Murthi, Mohamed Abumaree, Bill Kalionis

**Affiliations:** ^1^Department of Perinatal Medicine, Pregnancy Research Centre, The Royal Women’s HospitalParkville, VIC, Australia; ^2^Department of Obstetrics and Gynaecology, The University of MelbourneParkville, VIC, Australia; ^3^NorthWest Academic Centre, The University of MelbourneSt. Albans, VIC, Australia; ^4^College of Science and Health Professions, King Abdullah International Medical Research Center, King Saud bin Abdulaziz University for Health SciencesRiyadh, Saudi Arabia

**Keywords:** homeobox gene, transcription factors, placenta, angiogenesis, endothelial cells, macrovasculature, microvasculature

## Abstract

Homeobox genes are essential for both the development of the blood and lymphatic vascular systems, as well as for their maintenance in the adult. Homeobox genes comprise an important family of transcription factors, which are characterized by a well conserved DNA binding motif; the homeodomain. The specificity of the homeodomain allows the transcription factor to bind to the promoter regions of batteries of target genes and thereby regulates their expression. Target genes identified for homeodomain proteins have been shown to control fundamental cell processes such as proliferation, differentiation, and apoptosis. We and others have reported that homeobox genes are expressed in the placental vasculature, but our knowledge of their downstream target genes is limited. This review highlights the importance of studying the cellular and molecular mechanisms by which homeobox genes and their downstream targets may regulate important vascular cellular processes such as proliferation, migration, and endothelial tube formation, which are essential for placental vasculogenesis and angiogenesis. A better understanding of the molecular targets of homeobox genes may lead to new therapies for aberrant angiogenesis associated with clinically important pregnancy pathologies, including fetal growth restriction and preeclampsia.

## INTRODUCTION

Placental angiogenesis has become a focus for the development of diagnostic tools and potential therapeutics for pregnancy complications. Strategies for pro-angiogenic therapies are grounded on our knowledge of normal placental angiogenesis and our understanding of the angiogenic pathways that are disrupted in pregnancy pathologies. However, it is clear that our comprehension of normal angiogenesis in the placenta is lacking in comparison with other tissues and organs, such as the cardiovascular system. Furthermore, unique aspects of placental angiogenesis offer the potential for identifying novel angiogenic pathways from which new pro-angiogenic factors could be identified as potential therapeutics for various obstetric complications associated with aberrant angiogenesis. This review summarizes the genetic and molecular aspects of normal placental angiogenesis with a focus on placental endothelial cells. Our laboratory has major interest in understanding the transcriptional control of placental angiogenesis, with a specific focus on a family of transcription factors called “homeobox genes” and their expression in placental endothelial cells.

Homeobox genes play an essential role in regulating the function of vascular systems (Douville and Wigle, 2007). They coordinate the processes required for proper vascular formation during development, as well as the maintenance and repair of the vasculature systems throughout life. Often, homeodomain proteins work in concert within the vascular cells to achieve proper vessel function. Homeobox genes regulate the transcription of genes necessary for many vascular cell processes such as cell migration, invasion, proliferation, and tube formation. Several new downstream targets of specific homeobox genes have been identified in vascular systems in recent years. However, there are many homeobox genes that regulate angiogenesis where we have little or no knowledge of the biological pathways they regulate and their target genes of action. This review focuses on the expression of homeobox genes in placental vascular systems and their potential role in regulating placental angiogenesis.

## THE PLACENTA AND ITS VASCULATURE

**FIGURE 1:**
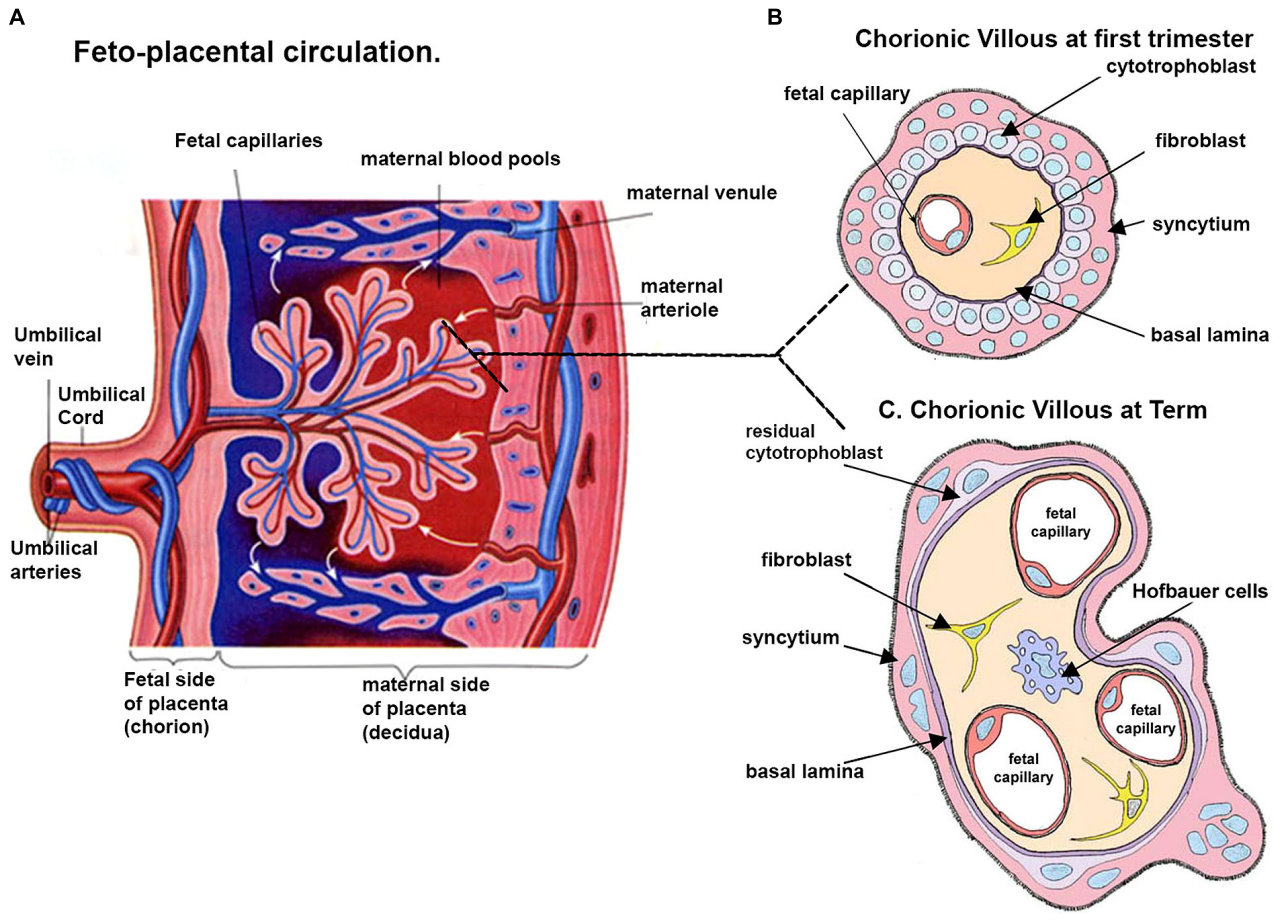
**Schematic representation of a human placenta. (A)** A representative drawing of the fetal placental circulation. Note the dotted line which shows the position from which drawings of a section through the chorionic villous at ~10 weeks **(B)** and term **(C)** are taken. **(B)** Chorionic villous the presence of syncytiotrophoblast, a layer of cytotrophoblast cells, connective tissue of the villus containing fibroblasts and the fetal capillaries. **(C)** At term, in some areas the placental membrane is so thin such that the syncytiotrophoblast comes into direct contact with the fetal capillary endothelium, and is thus called the vasculo-syncytial membrane. Adapted and modified from http://imueos.wordpress.com/2010/05/25/placenta-function/ and from http://php.med.unsw.edu.au/embryology/ASA_Meeting_2013_-_ Placenta

An efficient and high capacity materno-fetal exchange system is crucial for the growth and development of the fetus and the outcome of a healthy baby (Boyd and Hamilton, 1970). The placenta acts as a conduit between the maternal and fetal circulations and facilitates all gaseous and nutritive transfer between mother and fetus (Page, 1993; Moore and Persaud, 1998). This is achieved through a structural interface consisting of fetoplacental capillaries encased within terminal branches of the placental villous tree (i.e., the terminal villi), which are bathed in maternal blood perfusing into the intervillous spaces (see **Figure 1**; Gude et al., 2004). The metabolic needs of the fetus increase throughout pregnancy and the placenta adjusts to these demands through the continual development and adaptation of the placental villous vasculature thus ensuring sustained fetal growth and well-being (Chaddha et al., 2004).

In early placental development, villous vascularisation is preceded by trophoblast-mediated invasion of maternal uterine spiral arterioles, which establishes a maternal blood supply (Jaffe et al., 1997; Kingdom et al., 2000). The maternal blood filled lacunae then coalesce to create intervillous spaces, interposing pillars of trophoblasts, which gradually collapse and allow entry of blood from the uterine circulation (Lyall, 2005). The placental villous tree begins to form around day 13 post-conception, when remnants of the trophoblastic pillars proliferate into the intervillous spaces (Kingdom et al., 2000). A week later, vascularisation occurs by the *de novo* process of vasculogenesis (Risau, 1997; Kingdom et al., 2000). The villi are invaded by extraembryonic mesenchyme, which differentiates into endothelial and stromal support cells (Charnock-Jones et al., 2004). From these cells, a primitive placental vascular network is assembled and eventually connects with the embryonic circulatory system around day 32 post-conception (Kaufmann et al., 2004).

To perform the exchange functions required of it, the highly immature placental vasculature subsequently undergoes a phase of branching angiogenesis, which dramatically increases the number of villous blood vessels (Kaufmann et al., 2004). During this period, there is a corresponding rise in end-diastolic blood flow velocity, most likely reflective of a rise in fetal blood pressure (Hendricks et al., 1989). The increased villous capillary density improves fetoplacental blood flow to accommodate progressively increased fetal requirements (Ahmed and Perkins, 2000).

Around 26 weeks’ gestation, villous vascular development enters the final phase of non-branching angiogenesis, characterized by longitudinal growth of capillaries exceeding that of the villi themselves. The capillary loops bulge into the overlying villous trophoblasts, forming structures called terminal villi (Kingdom et al., 2000). Focal sinusoids, which are unique to the placenta because they possess a continuous endothelium and complete basal lamina, may also form in the fetoplacental capillaries, causing the outer vessel wall to be separated from maternal blood only by a very thin layer of syncytiotrophoblast called the vasculo-syncytial membrane (Burton and Tham, 1992). Terminal villus formation occurs exponentially during the third trimester (Chaddha et al., 2004). The end result of terminal villus formation is a dramatic increase in the surface area to volume ratio (Charnock-Jones, 2002; Chaddha et al., 2004) and the terminal villi form the major sites for diffusional exchange between the maternal and fetal circulations (Kingdom et al., 2000; Charnock-Jones et al., 2004; Kaufmann et al., 2004).

Therefore, the adaptation of the placental vasculature to increasing fetal demands follows two main strategies. Firstly, blood flow *per se* increases by lowering vascular impedance (Kaufmann et al., 2004). Branching angiogenesis initially creates parallel vessels of reduced mean length, and hence reduced impedance (Kaufmann et al., 2004). As capillaries lengthen due to non-branching angiogenesis, the sinusoids formed in them counterbalance the effect on total fetoplacental vascular impedance (Charnock-Jones, 2002). Secondly, the rate of diffusion across the placenta is improved by an increase in available surface area, and a reduction in villous membrane thickness; the vasculo-syncytial membrane separating maternal blood from fetal blood can be as thin as 1-2 μm (Charnock-Jones, 2002). Angiogenesis and the formation of terminal villi are the main processes that culminate in remodeling the placental vascular bed (Mayhew, 2003).

## IMPAIRED ANGIOGENESIS AND PREGNANCY-ASSOCIATED DISORDERS

Villous vascularisation is an important process in organogenesis and is essential for the placenta to function efficiently (Zygmunt et al., 2003). The spectrum of vascular defects associated with clinically significant pregnancy disorders attests to the close relationship between the placental vasculature and embryonic development. Compared with villi obtained from elective terminations, villi from placentae where intrauterine embryonic death and blighted ova was the outcome exhibit aberrant vascular characteristics manifest in significantly lower vascular density, fibrosis, and hydropic degeneration (Meegdes et al., 1988). Placentae from women with diabetes mellitus and gestational diabetes also show villous vascular maldevelopment and studies using light microscopy, electron microscopy and histochemical techniques have shown the length, diameter and surface area of fetoplacental capillaries to be increased (Jacomo et al., 1976; Jones and Fox, 1976; Mayhew et al., 1994). As well, some of the capillaries appear unduly immature (Kami and Mitsui, 1984).

Perhaps the most dramatic, best-characterized changes in the villous vasculature are seen in fetal growth restriction (FGR), which is a common and clinically significant disorder of pregnancy. FGR is defined as failure of the fetus to achieve genetically determined potential size to an extent where its health is adversely affected (Lin and Santolaya-Forgas, 1998). FGR affects 4–7% of live births in developed countries and contributes significantly to prematurity, perinatal morbidity, and mortality (Wang et al., 2007). Investigations using random block sampling and stereological studies reported reductions in the number, surface area, and volume of terminal villi in FGR-affected placentae, compared with placentae from uncomplicated pregnancies (Biagiotti et al., 1999; Egbor et al., 2006; Biswas et al., 2008; Vedmedovska et al., 2011; Almasry et al., 2012; Almasry and Elfayomy, 2012). Additionally, villous vessels exhibited fewer branches, and a majority of the vessels were slender and uncoiled (Teasdale, 1984; Teasdale and Jean-Jacques, 1988; Jackson et al., 1995; Chen et al., 2002; Mayhew, 2003; Tomas et al., 2010). A failure, or reduced capability, of branching angiogenesis in FGR is strongly associated (Kingdom et al., 2000) with a reduced supply of oxygen and nutrients to the fetus, and subsequent growth delay (Sanchez-Vera et al., 2005; Salafia et al., 2006).

Despite extensive research, it is unknown whether vascular changes cause FGR or whether these changes are a consequence of aberrant biological mechanisms in the FGR-affected placenta (Maulik, 2006; Maulik et al., 2006). Clearly, further research into the molecular regulation of angiogenesis in the placenta is vital.

## MOLECULAR REGULATION OF ANGIOGENESIS

**FIGURE 2:**
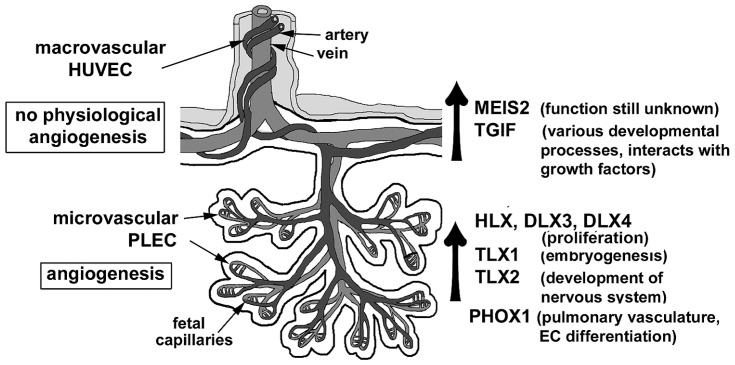
**A schematic diagram as illustrated describes the two distinct processes of vasculogenesis and angiogenesis involved in fetoplacental vascular development in human pregnancy. Adapted and modified from** Murthi et al. (2008).

Angiogenesis involves distinct changes in the phenotype of endothelial cells, the central cellular organizational units of vascular structures. **Figure 2** shows the two distinct processes of vasculogenesis and angiogenesis involved in fetoplacental vascular development in human pregnancy.

In a multi-step event, quiescent endothelial cells are first activated to re-enter the cell cycle (Myers et al., 2002). As a consequence of increased cellular proliferation, proteolytic enzyme production is up-regulated in order to degrade the basement membrane. The endothelial cells then migrate into the surrounding stroma and gradually assemble into a tube-like capillary structure with a patent lumen. After a new basement membrane is synthesized, pericytes are recruited to the outside of the new capillary to complete the formation of a stable, quiescent vessel (Sato, 2000).

The stimuli for these complex, temporally coordinated changes are communicated from the microenvironment surrounding the endothelial cell surface to the nucleus through multiple signaling pathways (Patel et al., 2005). At the molecular level, the growth factors and receptors that activate these pathways have been extensively studied *in vitro and in vivo* (Arderiu et al., 2007; Winnik et al., 2009). Vascular endothelial growth factor (VEGF), placental growth factor (PlGF), and the angiopoietins are considered the most influential factors (Patel et al., 2005). The primary receptors for VEGF are VEGF receptor-1 (VEGFR-1) and VEGF receptor-2 (VEGFR-2), while PlGF only binds to VEGFR-1 (Patel et al., 2005). VEGF has been demonstrated to be a potent stimulator of endothelial cell proliferation, migration, and production of plasminogen activators required for basement membrane digestion (Regnault et al., 2003; Escudero et al., 2014). Studies of chicken chorioallantoic membranes have shown that VEGF binding to both VEGFR-1 and VEGFR-2 results in branching angiogenesis, while PlGF binding to VEGFR-1 alone mediates non-branching angiogenesis (Wilting et al., 1996). The angiopoietin family comprises two main factors, angiopoietin-1 (Ang-1) and angiopoietin-2 (Ang-2) and are both antagonistic ligands of a common receptor, the tyrosine-kinase with immunoglobulin-like and epidermal growth factor-like domains-2 (TIE-2). While Ang-1 binding to TIE-2 promotes vascular stabilization, Ang-2 allows vessels to remain in a more plastic state (Kurz et al., 1998).

In normal pregnancies, placental expression of important growth factors correlates with their established roles. For example, expression of VEGF and VEGFR-2 is highest during early gestation, which coincides with vasculogenesis and branching angiogenesis, but expression declines with advancing pregnancy (Jackson et al., 1994). Conversely, PlGF and VEGFR-1 expression is highest toward term, coinciding with non-branching angiogenesis (Clark et al., 1996). A concurrent decrease in Ang-2 expression, and increase in Ang-1 expression at the end of the second trimester, is believed to mediate the transition from branching to non-branching angiogenesis (Geva et al., 2002).

Our knowledge of the molecular regulation of angiogenesis in the placenta is incomplete. Nuclear transcription factors integrate upstream signals generated by the binding of growth factors to their receptors. Transcription factor binding to specific DNA sequences within the promoter regions stimulates or represses expression of batteries of downstream target genes (Hamik et al., 2006). Transcription factors are considered to be the master regulators that determine gene expression profiles that culminate in the activated, angiogenic phenotype. Loss of function studies clearly demonstrate that transcription factors including *TBX4*, *CDX2, CDX4, HAND1, FOXF1, CITED2* are required for placental development (Mahlapuu et al., 2001; Cross et al., 2002; Naiche and Papaioannou, 2003; Cross, 2006; Preis et al., 2006; van Nes et al., 2006). However, the target genes regulated by these transcription factors are largely undefined. Recent studies have provided evidence for transcriptional control of VEGF signaling by Notch ligand as well as hypoxia-inducible factor (*HIF1a*) in placental angiogenesis (Fang et al., 2013). Morphological and phenotypic analyses of the human placenta using whole mount immunofluorescence technique were employed to demonstrate that early human placental blood vessels express high levels of the pro-angiogenic receptors VEGFR1, VEGFR3 and the activated signal transduction and activator of transcription 3 (pSTAT3) suggesting that these molecules play a role in regulation of placental vascular development (Bushway et al., 2014). Thus, an understanding of transcriptional mechanisms would afford a valuable insight into the downstream angiogenic signaling cascades in the placenta, which as yet, remain largely unexplored in the human placenta.

## HOMEOBOX GENES

A particular large family of transcription factors that provides a fertile area for studying placental angiogenesis is the homeobox gene family. Characterized by a common 60-amino-acid DNA-binding motif known as the homeodomain, and homeobox genes were first identified in *Drosophila* through investigations of mutations that gave rise to homeotic transformations (McGinnis and Krumlauf, 1992). Subsequently, it was discovered that three-dimensional patterning and body plan formation during embryogenesis are largely attributable to action of homeobox genes, due to their capacity to spatiotemporally regulate the basic processes of differentiation, proliferation, and migration (Manley and Levine, 1985; Han et al., 1989). Homeobox genes can regulate genes responsible for cell adhesion, migration, proliferation, growth arrest, and the expression of cytokines needed for extracellular matrix interactions (Graba et al., 1997; Svingen and Tonissen, 2006; Hueber et al., 2007) all of which are functions characteristic of the angiogenic phenotype.

## HOMEOBOX GENES IN ANGIOGENESIS

Evidence in the literature increasingly supports a substantial role for homeobox genes in general vascular development, and particularly in endothelial cell function (Gorski and Walsh, 2000, 2003; Gorski and Leal, 2003; Douville and Wigle, 2007). A well-known example is *Gax*, a homeobox gene originally isolated from a rat aortic cDNA library, which is widely expressed in embryonic muscle precursors (Gorski et al., 1993). Initial investigations of the human homolog, *Gax*, were conducted primarily on vascular smooth muscle cells, where *Gax* was shown to induce G_1_ cell cycle arrest and reduce cell migration (Witzenbichler et al., 1999). Gorski and Leal (2003) subsequently confirmed *GAX* expression in endothelial cells using immunohistochemical methods on sections of highly capillarised human kidney. Succeeding *in vitro* investigations revealed that *GAX* prevented VEGF-induced endothelial cell migration and tube formation through the repression of multiple genes involved in the pro-angiogenic nuclear factor kappa-beta (NFκ-B) signaling pathway (Patel et al., 2005). Hence, *GAX* emerged as an important inhibitor of the angiogenic phenotype. Other less well studied homeobox genes are implicated as positive regulators of angiogenesis. For example, human *HOXA9* promotes endothelial cell migration, in part by activating the expression of EphB4; a receptor tyrosine-kinase that shows increased expression in tumor-induced vascularisation (Bruhl et al., 2004).

*HoxA9*^-/-^ mouse embryos display a poorer angiogenic response to hypoxia and have decreased numbers of endothelial cell precursors (Rossig et al., 2005). Complementary pro-angiogenic functions have also been described for the paralogous homeobox genes *HoxD3* and *HoxB3*. *HOXD3* not only promotes endothelial cell invasion of the extracellular matrix early in angiogenesis, but also regulates the subsequent capillary morphogenesis of these new vascular sprouts (Douville and Wigle, 2007). Although these findings generally emphasize the multifaceted importance of homeobox genes in angiogenesis, the studies were conducted within the context of embryonic development and/or tumor-induced adult neovascularisation.

Studies by Shaut et al. (2008) have reported that *HoxA13* is essential for placental vascular patterning and labyrinth endothelial specification. In the absence of *HoxA13* function, placental endothelial morphology is altered causing a loss in vessel wall integrity, edema of the embryonic blood vessels and mid-gestational lethality. The authors have also reported on the novel transcriptional program by which *HoxA13* directly regulates *Tie2* and *Foxf1* in the placental labyrinth endothelia, providing a functional explanation for the mid-gestational lethality exhibited by *HoxA13* mutant embryos. However, homeobox gene contribution(s) to extraembryonic angiogenesis, particularly in the human placenta, remains largely unexplored.

## HOMEOBOX GENES IN THE PLACENTA

Currently, information about the role of homeobox genes in placental tissues is mainly derived from studying mouse gene knockouts (Rossant and Cross, 2001). For example, targeted deletion of *Esx1* (Fohn and Behringer, 2001) and *Dlx3* (Morasso et al., 1999) resulted in disruption of the vascular network in the placental labyrinthine layer, which in mice is thought to be functional equivalent of the human placental villi (Cross et al., 2003a, b). Not only were embryos in both cases growth-restricted, but failure to establish an adequate placental circulation in *Dlx3*^-^^/^^-^ mutants resulted in embryonic lethality (Morasso et al., 1999). Together, these studies provide genetic proof that homeobox genes are not only regulators of placental organogenesis but they are also specific regulators of placental vascular development. Furthermore, homeobox genes can directly or indirectly influence fetal viability. We carried out the first screening of a 32-week placental cDNA library for homeobox genes, which led to the isolation of *DLX4, MSX2, GAX*, and *HLX* (Quinn et al., 1997; Rajaraman et al., 2008). Immunohistochemical analyses identified the localisation of these homeobox genes in both trophoblasts and endothelial cells of the human placenta (Murthi et al., 2006a; Rajaraman et al., 2008; Chui et al., 2010).

## HOMEOBOX GENE EXPRESSION IS ALTERED IN HUMAN FETAL GROWTH RESTRICTION CHARACTERIZED BY IMPAIRED PLACENTAL ANGIOGENESIS

Using a clinically well-defined cohort of idiopathic FGR (*n* = 25) and gestation-matched control (*n* = 25) pregnancies, we reported an overall decrease in homeobox gene *HLX* and *ESX1L* expression in all cell types, including endothelial cells, in FGR-affected placentae compared with GMC (Murthi et al., 2006a, b). Subsequently, we also reported that homeobox genes *DLX4* and *DLX3* showed increased expression in FGR-affected placentae (Murthi et al., 2006c; Chui et al., 2012), whereas *GAX* and *MSX2* showed no significant difference. Our studies represented the most comprehensive and extensive analyses of homeobox genes in placental pathologies undertaken. *In situ* mRNA hybridisation and immunohistochemical studies on placental sections localized the expression of these genes not only to placental trophoblasts but also to endothelial cells that comprise the fetal capillaries (Quinn et al., 1998a, b, 2000).

## HOMEOBOX GENE EXPRESSION IN PLACENTAL ENDOTHELIAL CELLS

At least two functionally distinct endothelial cell types, macrovascular and microvascular exist within the human placenta (Lang et al., 1993; Ugele and Lange, 2001). Macrovascular endothelial cells [human umbilical vein endothelial cell (HUVEC)] line the large conduit vessels of the umbilical cord and isolated cells from the vein have been used extensively to model vasculogenic and angiogenic processes occurring in tissues such as the placenta (Demir et al., 1989; Wang et al., 2004). Microvascular endothelial cells vascularise the cotyledons of the placenta. It is important to study the microvascular environment of the placenta because in placental disorders such as FGR and PE, structural and vascular changes occur within the microvasculature of the terminal villi that impact on maternal-fetal gas and nutrient exchange (Demir et al., 1989; Kingdom et al., 2000; Dye et al., 2004; Wang et al., 2004). Lang et al. (2003) have reported that distinct morphogenetic, antigenic, and functional characteristics exist between microvascular and macrovascular endothelial cells of the human placenta and demonstrated differences in the secretion of vasoactive substances and the proliferative response to cytokines between microvascular and macrovascular endothelial cells of the human placenta. The different reactions of microvascular and macrovascular endothelial cells to various stimuli (Lang et al., 2003) are likely to reflect differences in the activation of transcription factors that mediate signal transduction mechanisms in the two cell types.

In addition, several studies have found various morphological, antigenic, growth, and functional differences between the two endothelial cell types in association with pathological conditions (Thorin and Shreeve, 1998; Charnock-Jones et al., 2004; Pollheimer and Knofler, 2005). Therefore, it was concluded that isolated microvascular endothelial cells from the chorionic villi have advantages as a model to study placental vascular development over macrovascular HUVEC.

To isolate and enrich placental microvascular endothelial cells (PLEC), we used a modified methodology based on the perfusion-based technique described by Lang et al. (2003). After cannulation of the chorionic vessels and removal of fetal blood, Lang et al. (2003) introduced proteolytic enzymes into the perfused cotyledon in a specific volume of buffer that was perfused into the placenta at a variable flow rate by employing a gravity feed system. In contrast, our modified methodology involved pumping the enzymes into the vasculature at a constant flow rate for a variable length of time and until no further venous outflow was obtained. This modified technique achieved a controlled delivery of the enzymes and an enriched population of PLEC (Murthi et al., 2007).

Freshly isolated PLEC were used to identify the homeobox genes expressed in the placental microvasculature, and expression of homeobox genes was compared with that of macrovascular HUVEC. Conventional reverse transcriptase polymerase chain reaction (PCR) was used to detect mRNA levels of homeobox genes *DLX3, DLX4, MSX2, GAX*, and *HLX* (formerly known as *HLX1* or *HB24*) in both PLEC and HUVEC. Our study was the first to show *DLX3, DLX4*, and *MSX2* are expressed in macrovascular HUVEC. We also reported that the mRNA levels of *HLX* mRNA in HUVEC were significantly lower compared with PLEC (Murthi et al., 2007). These data provided further evidence of heterogeneity in homeobox gene expression between microvascular PLEC and macrovascular HUVEC, which most likely reflects significant differences in endothelial cell function in the two different cellular environments.

*HLX* is important in the proliferation and lineage commitment of haematopoietic cells (Deguchi et al., 1992). In the human placenta, *HLX* mRNA expression is restricted to proliferating cell types such as villous cytotrophoblast and extravillous cytotrophoblast cells in the proximal regions of the invading cell columns (Rajaraman et al., 2008). In our study, we showed *HLX* mRNA expression in placental endothelial cells (Murthi et al., 2007), which are also proliferative cell types. Microvascular endothelial cells of the placenta, particularly within the terminal and intermediate villi of term placentae, have a higher level of proliferative activity in comparison with their macrovascular counterparts (Murthi et al., 2007). Moreover, in response to PlGF, PLEC have a significantly greater proliferative activity compared with HUVEC (Lang et al., 2003). *HLX* levels in PLEC are relatively higher than in HUVEC, and PLEC have greater proliferative potential. Taken together, these data suggests a possible role for *HLX* in the proliferative capacity of microvascular endothelial cells. The transcriptional regulation of proliferation, migration, and invasion of PLEC by homeobox genes *DLX3, DLX4, MSX2, GAX*, and *HLX* is yet to be explored.

The reported the co-expression of *HLX, MSX2, GAX*, and *DLX4* in PLEC (Murthi et al., 2007) and this specific combination of homeobox genes may be important in mammals. For example, *Hlx* is co-expressed with members of the *Msx, Gax (also known as Mox)* and *Dlx* families in the mouse embryo. *HLX* is expressed with *MSX2, GAX* (also called *MOX2*) and *DLX4* in the trophoblast cell layers of the human placenta (Quinn et al., 1998b). Quinn et al. (2000) predicted that the combination of homeobox genes could play a significant role in the regulation of epithelial–mesenchymal cell interactions in the extraembryonic tissues. Therefore, co-expression of the homeobox genes in both trophoblast and endothelium may be important in the coordination of villous outgrowth and angiogenesis that is seen in the terminal villi and is essential for the efficient functioning of the placenta as it grows. Thus, our study on the homeobox gene expression profiling in placental endothelial cells (Murthi et al., 2007) further emphasized the importance of studying microvascular endothelial cells (i.e., PLEC) as a model for the placental microvascular bed, or other microcirculation systems.

## IDENTIFICATION OF NOVEL HOMEOBOX GENES IN PLACENTAL ENDOTHELIAL CELLS

To further expand our knowledge of the repertoire of homeobox genes expressed in placental endothelial cells, in a subsequent study by Murthi et al. (2008) we carried out microarray expression profiling on endothelial cells and analyzed public microarray expression profile databases. We have employed PCR and real-time PCR methods to corroborate the microarray data and to compare relative expression levels of homeobox genes in PLECs and HUVEC.

Microarray expression data as reported in Murthi et al. (2008) suggested that novel homeobox genes are expressed in microvascular placental endothelial cells. These homeobox genes, *HEX, PHOX1, LIM6, HOX*B7 and *TGIF*, have not been previously detected in the placenta and were selected because they exhibited the greatest relative expression in the microarray data (Murthi et al., 2008). Novel homeobox genes *TLX1* and *TLX2* homeobox gene expression data was obtained from the GNF Microarray Analysis Data for the Human U95A microarray, Version 2 dataset (http://expression.gnf.org; Su et al., 2002). Homeobox genes *LIM6, HOXB7, TGIF, PHOX1*, and *HEX* were expressed in the endothelial cells of the placenta.

Expression of these homeobox genes in the placenta or in placental endothelial cells has not been previously reported. *HEX* (Nakagawa et al., 2003), *PHOX1/Prx1* (Ihida-Stansbury et al., 2004), and *HOXB7* (Care et al., 2001) homeobox genes have been previously described in endothelial cells from various sources but have not been described in any endothelial cell type and may represent novel endothelial regulatory genes. Validation of high-throughput gene microarray screening data of potentially novel homeobox gene expression in endothelial cells is essential. In our study (Murthi et al., 2008), the microarray data were further corroborated by independent methods such as RT-PCR and real-time PCR. Thus, our study was the first to demonstrate that the novel homeobox genes *TLX1, TLX2, PHOX1, MEIS2*, and *TGIF* are expressed in PLEC. In addition, in the same study, we have also reported a differential expression of *TLX1, TLX2, PHOX1, MEIS2*, and *TGIF* mRNA levels in macrovascular and microvascular endothelial cells.

Thus, we have identified novel homeobox genes in microvascular endothelial cells, and consistent with our previous studies reported in Murthi et al. (2007), we have shown that homeobox genes are differentially expressed between micro- and macrovascular endothelial cells. Our studies also provided further evidence of heterogeneity in homeobox gene expression between PLEC and HUVEC, which reflects the significant differences in endothelial cell function in the two different cellular environments.

**FIGURE 3:**
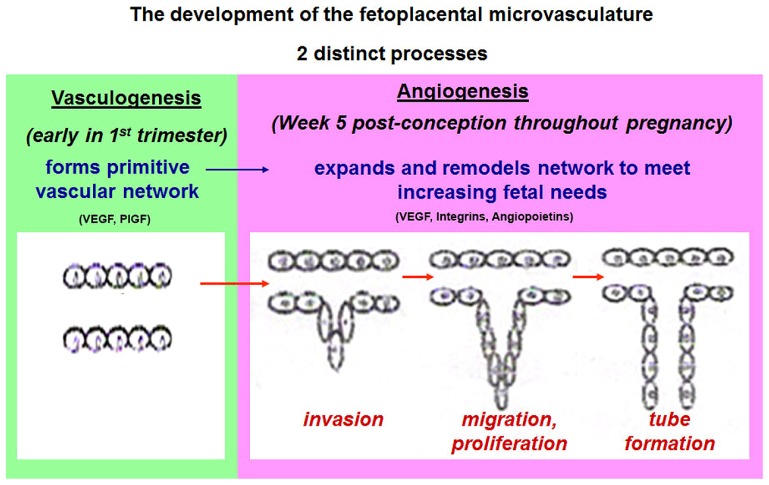
**A schematic representation of the distinct endothelial cell types in the human placenta**. Differential expression of novel homeobox genes *TGIF* and *MEIS2* in macrovascular HUVEC and *HLX*, *DLX*3, *DLX4*, *TLX1*, *TLX2*, and *PHOX1* in freshly isolated placental endothelial cells (PLECs) are summarized. Figure adapted from Sato (2000), Kingdom et al. (2000).

In summary, our studies have reported homeobox genes that are novel not only in placental microvascular endothelial cells but also in the macrovascular endothelial cells of the placenta (Murthi et al., 2008). **Figure 3** summarizes the association between the detection of homeobox gene expression and the regions of angiogenic potential in the human placenta. In the microvasculature, where angiogenesis is predominant, we have identified increased expression of homeobox genes *TLX1*, *TLX2*, and *PHOX1*. In the macrovasculature, where there is limited angiogenesis, the level of *TGIF* and *MEIS2* are significantly increased suggesting that the heterogeneity in homeobox gene expression between PLEC and HUVEC that could reflect differences in the angiogenic potential in the two different endothelial environments. Functional studies in cultured endothelial cells are underway in our laboratory to determine the role of novel homeobox genes.

## DOWNSTREAM TARGETS OF HOMEOBOX GENES

Homeobox genes control transcription by binding to regulatory elements in the promoter regions of target genes. Miano et al. (1996) first reported the expression of several homeobox genes in the cardiovascular and lymphatic vasculature. More recently, several homeobox genes were shown to affect processes in embryonic and adult tissues, including angiogenesis and wound healing (Kachgal et al., 2012). Homeobox genes activate either growth or migration of vascular cells to promote angiogenesis or wound healing or restore and maintain quiescent differentiated tissue function by modulating the expression of pro-angiogenic or anti-angiogenic factors. **Table 1** provides examples of downstream target genes of homeobox genes that are required for the regulation of endothelial function in general.

**Table 1:** Examples of target genes downstream of homeobox genes required for the regulation of endothelial functions.

Homeobox genes	Target genes	Regulation	EC function	Reference
**Pro-ang0iogenic**				
HOXA3	uPAR	+	Migration	Mace et al. (2005)
HOXA9	MMP-14	+	MigrationProliferationActivation	Bruhl et al. (2004)
	EphB4	+		Rossig et al. (2005)
	eNOS	+		
	VEGFR2	+		
HOXB5	VEGFR2	+	Activation	Wu et al. (2003)
HOXB3	Ephrin A1	+	Vessel formation	Myers et al. (2000)
HOXD3	Collagen A1	+	Adhesion and migration	Boudreau and Varner (2004)
MEOX2	MLLT7	-	Apoptosis	Wu et al. (2005)
PROX1	Cyclin E1	+	Proliferation	Petrova et al. (2002)
**Anti-angiogenic**				
HHEX	VEGFR2	-	Activation	Nakagawa et al. (2003)
HOXD10	FGF2	+	Recruitment	Chen et al. (2009)
HOXA5	VEGFR2	+	Adhesion	Arderiu et al. (2007)
	Ephrin A1			
	*HIF1a*			
MEOX2	P21	+	Cell cycle arrest	Gorski and Leal (2003)

Studies using umbilical or uterine artery Doppler for identifying FGR, in the absence of maternal hypertensive disease, show that maternal serum sFLT-1 is increased in these pregnancies compared with pregnancies of normotensive women delivering average for gestational age infants (Crispi et al., 2006; Stepan et al., 2007; Wallner et al., 2007; Chaiworapongsa et al., 2008, 2013). More recent studies by Borras et al. (2014) have reported that maternal plasma free VEGF (f-VEGF) and s-Flt-1 were significantly higher in FGR compared with controls and the f-VEGF/sFlt-1 quotient was significantly lower in the FGR group compared with controls. Although the VEGF family has important roles in normal and complicated pregnancies, the current predictive value of the VEGF family as biomarkers appears to be limited to early onset preeclampsia (Andraweera et al., 2012).

Studies from our laboratory, using a real-time PCR-based gene profiling, recently identified candidate target genes of homeobox gene *DLX3* as regulators of trophoblast differentiation; *GATA2* and *PPAR*γ (Chui et al., 2013). The expression of *GATA2* and *PPAR*γ were further assessed in placental tissues and showed increased expression in FGR-affected tissues compared with gestation-matched controls. Our studies showed that *DLX3* orchestrates the expression of multiple regulators of trophoblast differentiation and that expression of these regulatory genes is abnormal in FGR.

Xin et al. (1999) have shown that *PPAR*γ ligands suppress *VEGFR1* and *VEGFR2* expression in HUVECs. Anti-angiogenic actions of 15-lipoxygenase on angiogenesis is regulated by *PPAR*γ and *VEGF* by inhibiting the expression of *VEGFR2* in endothelial cells (Mochizuki and Kwon, 2008; Viita et al., 2008). Because the chemical *PPAR*γ ligands thiazolidinediones have been used widely for the treatment of type 2 diabetic patients, many of whom experience vascular diseases, clarifying the precise role of *PPAR*γ in defective placental angiogenesis may be of clinical significance.

**FIGURE 4:**
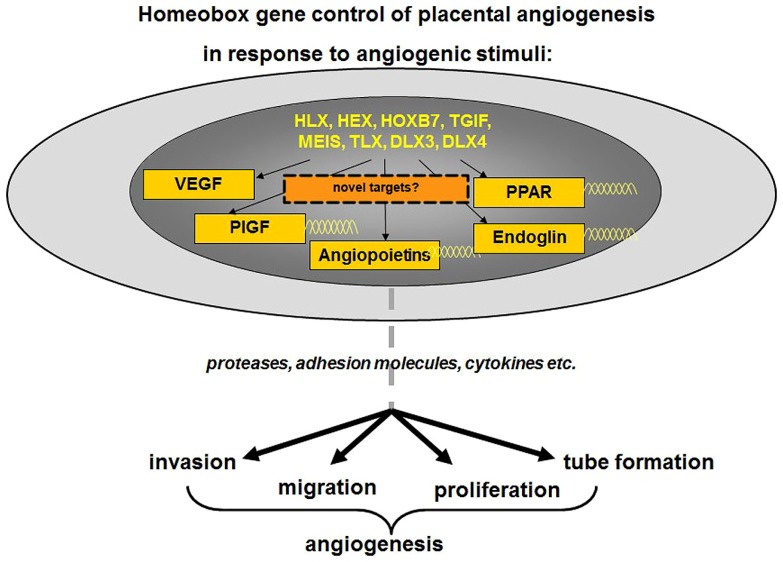
**As depicted, in response to angiogenic stimuli, altered expression of homeobox genes in the placental microvascular endothelial may directly or indirectly alter the expression of angiogenic molecules**. Further analyses of downstream targets may reveal novel angiogenic markers. These angiogenic molecules in turn may regulate genes responsible for cell adhesion, migration, proliferation, growth arrest, and the expression of cytokines needed for extracellular matrix interactions all of which are functions characteristic of the angiogenic phenotype. Figure adapted and modified from Sato (2000).

Current studies in our laboratory are also focused on identifying target genes of homeobox genes *TGIF, MEIS, HOXB7*, and *HHEX* in human placental endothelial cells, which may reveal molecular pathways responsible for fundamental cellular functions such as endothelial cell migration, invasion, proliferation, and tube formation that are important for placental angiogenesis. As depicted in **Figure 4**, in response to angiogenic stimuli or insult, as in the case of FGR or PE, altered expression of homeobox genes in the placental microvascular endothelial may directly or indirectly alter the expression of angiogenic molecules. These angiogenic molecules in turn may regulate genes responsible for cell adhesion, migration, proliferation, growth arrest, and the expression of cytokines needed for extracellular matrix interactions all of which are functions characteristic of the angiogenic phenotype.

## CONCLUSION

Clearly, identifying target genes regulated by homeobox genes in placental microvascular endothelial cells will reveal the biological pathways regulated by homeobox genes. These pathways will provide important information on the function of homeobox genes in placental angiogenesis. Although homeobox gene nuclear transcription factors are unlikely to be ideal disease biomarkers or therapeutic targets, their target genes, if secreted, may provide viable biomarkers or diagnostic markers. A better understanding of cellular and molecular mechanisms that regulate homeobox genes in placental endothelial cells may lead to new approaches for correcting aberrant angiogenesis observed in pregnancy pathologies, including FGR and preeclampsia.

## Conflict of Interest Statement

The authors declare that the research was conducted in the absence of any commercial or financial relationships that could be construed as a potential conflict of interest.
